# Positive affect as coercive strategy: conditionality, activation and the role of psychology in UK government workfare programmes

**DOI:** 10.1136/medhum-2014-010622

**Published:** 2015-06

**Authors:** Lynne Friedli, Robert Stearn

**Affiliations:** 1 London, UK; 2 Department of English and Humanities, School of Arts, Birkbeck, University of London, London, UK

**Keywords:** Politics

## Abstract

Eligibility for social security benefits in many advanced economies is dependent on unemployed and underemployed people carrying out an expanding range of job search, training and work preparation activities, as well as mandatory unpaid labour (workfare). Increasingly, these activities include interventions intended to modify attitudes, beliefs and personality, notably through the imposition of positive affect. Labour on the self in order to achieve characteristics said to increase employability is now widely promoted. This work and the discourse on it are central to the experience of many claimants and contribute to the view that unemployment is evidence of both personal failure and psychological deficit. The use of psychology in the delivery of workfare functions to erase the experience and effects of social and economic inequalities, to construct a psychological ideal that links unemployment to psychological deficit, and so to authorise the extension of state—and state-contracted—surveillance to psychological characteristics. This paper describes the coercive and punitive nature of many psycho-policy interventions and considers the implications of psycho-policy for the disadvantaged and excluded populations who are its primary targets. We draw on personal testimonies of people experiencing workfare, policy analysis and social media records of campaigns opposed to workfare in order to explore the extent of psycho-compulsion in workfare. This is an area that has received little attention in the academic literature but that raises issues of ethics and professional accountability and challenges the field of medical humanities to reflect more critically on its relationship to psychology.

## Introduction

Negativity enacts the dissent without which politics disappears. Negativity, in this sense, is inseparable from the struggles of subordinated persons to resist the social conditions of their devaluation (p.xii).^1^

Three people start today on this ‘work experience’. They are to help us for up to 30 hours a week for eight weeks over the Christmas period. I am terrified by the idea that head office think they don’t need to pay their staff. I myself am on part time minimum wage and if they can have workers for free now, what is to stop them making my position redundant and using job centre people to run the store at no cost to themselves. (Shoezone employee, November 2012)^2^

The cajoling of individuals into a positive affect and ‘motivated’ stance with regard to their own subordination.^3^

This paper considers the role of psychology in formulating, gaining consent for and delivering neoliberal welfare reform, and the ethical and political issues this raises. It focuses on the coercive uses of psychology in UK government workfare programmes: as an explanation for unemployment (people are unemployed because they have the wrong attitude or outlook) and as a means to achieve employability or ‘job readiness’ (possessing work-appropriate attitudes and beliefs). The discourse of psychological deficit has become an established feature of the UK policy literature on unemployment and social security and informs the growth of ‘psychological conditionality’—the requirement to demonstrate certain attitudes or attributes in order to receive benefits or other support, notably food.^i^ In addition, positive affect is routinely imposed in workfare programmes via the content of mandatory training courses and through job centre or contractor ‘messaging’, for example, motivational tweets or daily positive emails to claimants.^5^

The role of workfare in regulating labour through enforcing low-paid, insecure work—‘creating workers for jobs that nobody wants’—has been widely debated, frequently in connection with increased welfare conditionality.^6^ This literature notes that eligibility for various benefits is now dependent on unemployed and underemployed people carrying out an expanding range of job search, training and work preparation activities, as well as mandatory unpaid labour.^4^ ^7^ Our focus on workfare schemes and interventions targeting unemployed people’s attitudes is also indebted to the body of feminist and Marxist critical work on emotional and affective labour.^8^ ^9^ However, the concerns of this literature—the management and suppression of feeling in service work and the hire of subjectivity in cognitive and affective labours; the constitutive, personality-forming effects of both—differ from ours.^10^ The personality set to work is not the same as the personality seeking employment. What the Jobcentre requires is a good but not particular attitude to work in the abstract and a capacity for adaptability that has no object. As a jobseeker you are required to accept that what differentiates you, the failed and undeserving jobseeker, from other more deserving and successful jobseekers is a set of attitudes and emotional orientations. The aim is not a job, but the generic skill, attribute or disposition of employability.^11^ Focusing on this aspect of governance, there has also been extensive critical attention paid to ‘the psyche as a site of power and object of knowledge’ (p.iii),^12^ and, under the rubric of the government of the self,^13^ ^14^ to the role of strengths-based discourse in the formation of systems of discipline and control and the formulation of active welfare subjectivities.^15^ ^20^

However, there has been a marked silence about the use and misuse of psychology in public policy on many fronts: especially, the role of psychological institutions and professions in workfare and in the emerging employment services industry; and the coercive and punitive nature of many psycho-policy interventions. The voices of claimants and the disadvantaged and excluded populations who are the primary targets of these enforced programmes are little heard. So, this paper is also an effort to challenge that silence: we aim to stimulate more critical reflection on the relationship of medical humanities to psychology and the wider ‘well-being’ field, and to generate greater debate about professional accountability for these developments.^ii^ We draw on personal testimonies of people experiencing workfare,^iii^ UK policy and document analysis, and social media records of the activity of campaigns opposed to workfare.

## Conditionality

In the last three decades, welfare reforms in many rich democratic states have led to increased emphasis on the conditionality of social security payments and the ‘activation’ of their recipients, avowedly to avert or correct ethical and psychological ‘dependency’ and other forms of debility, depression and etiolated work ethic,^12^ ^13^ ^20^ ^22^ ^23^ which are widely thought to be both symptom and cause of unemployment.^24^ Failure to meet conditions placed on eligibility for benefits is punished directly by benefit sanctions (the part or total cessation of social security payments for a given period of time),^25^ as well as indirectly by compulsory ‘support’ in the form of workfare, ‘skills training’, psychological referral or psychometric testing. The conditions are diverse in kind as well as wide-ranging: from age and residence criteria, or restrictions on numbers of (paid) hours worked per week, to possession of certain levels of qualifications and the capacity to demonstrate positive opinions on employment.^26^ The expansion of conditionality in this way is linked to the continually increasing rate at which Jobseeker's Allowance (JSA) and Employment and Support Allowance claimants are sanctioned (the three months to September 2013 saw JSA claimants sanctioned at a rate of 6% of claimants per month, the highest since the introduction of JSA in 1996).^27^ Failure to participate in a training or employment scheme is the most frequently occurring ‘failure’ that results in a sanction. These mandatory interventions designed to ‘shift attitudes and beliefs’ have become an important element of ‘activating’ the unemployed, and are the focus of this paper.^4^ ^12^ ^15^ ^20^ Although payments by the state to people without jobs have been tied to desirable patterns of behaviour since their first institution,^22^ ^23^ ^28–31^ the unemployment policies of reformed welfare states now aim at more complete and intimate behaviour change through coercive mechanisms of greater scope.^32^

The reorganisation of welfare in the UK accompanying current moves to replace six working-age benefits with Universal Credit (UC) by 2017 is the latest face of this broader trend.^22^ ^33^ Under UC, each claimant will be issued with a Claimant Commitment (CC) (which has already replaced the Jobseeker's Agreement for new claimants at many Jobcentres). The CC enables Jobcentre staff to check claimants’ behaviour against the range of ‘work-related requirements’ to which they have committed.^34^ These requirements are sorted into a tiered system of conditionality. UC furthers the Department for Work and Pensions’ (DWP) project of personalised behavioural conditionality,^35^ of which psychological coercion and governance—imposed in and through workfare—is an integral part. For the first time, under UC, these forms of conditionality are extended to claimants also in work.^36^

## Workfare

By workfare we mean the ‘work-for-your-benefits’ schemes in which unemployed people are forced to work for a charity, business, social enterprise, public service or government agency in order to continue to be eligible for benefits. We also include the range of skills-building and motivational workshops that are presented alongside such schemes—as part of a range of activities that unemployed people are obliged to undertake—and schemes that are composed of training courses in tandem with unpaid work (Skills Conditionality is an example of the former; Traineeships and Sector-Based Work Academies of the latter). The participation of unemployed people in schemes with training elements is secured by the same means as work placement schemes: through the threat—tacit or explicit, indirect or direct—of sanctions. It is important that they be looked at as a group (and that we adopt a definite but not too narrow definition of workfare) since this is both how they are implemented and how they impact on unemployed people.

In the UK, as in many other Western states, workfare is organised within an employment services sector that extensively contracts out services to for-profit and non-profit organisations.^37^ Where UK policy differs is in the commissioning practices of DWP, which has outsourced the procurement, design and arrangement of employment services and unpaid work placements to a small number of large-scale for-profit companies.^38^ The Jobcentre refers a claimant to a ‘prime’ contractor (Ingeus, A4e, G4S, Serco) that provides some services and mandatory forms of assistance and contracts out others to smaller contractors, which arrange unpaid work placements at charities and businesses. Government contracts specify little about the details of the services to be provided: what control there is, government exerts through a tiered system of ‘payment by results’.^37^ ^39^ ^40^ The fact that most psycho-compulsion occurs within this ‘black box’ has important implications since there is virtually no oversight of the content of such compulsions, no professional accountability and no effective means of appeal against them.^41–43^

Workfare is central to normalisation of the idea that harsh sanctions should be used to underwrite certain obligations of citizenship, and to singling out as the paramount obligation the enforcement of work, with no regard to the specific character of that work or to a person's other responsibilities.^13^ ^14^ Workfare furthers the separation of work and livelihood and normalises the idea that certain groups of people are not entitled to payment for their labour and that lengthy periods of unpaid labour (eg, internships or ‘volunteering’) are a precondition for employment. In this way, it undermines the security, pay and conditions of all workers and non-workers.^44^ Moreover, it demands that people assent to the idea that paid work as it is currently organised is the only route to both personal fulfilment and public value and obscures the economic reality of a dual labour market that produces and relies upon the stratification of work and the escalating inequalities in income and quality of working life.^45^ ^46^

## Psycho-compulsion

Psycho-compulsion, defined as the imposition of psychological explanations for unemployment, together with mandatory activities intended to modify beliefs, attitude, disposition or personality, has become a more and more central feature of activating the unemployed and hence of people’s experience of unemployment. There has been little debate about the recruitment of psychology—and, by implication, psychologists—into monitoring, modifying and punishing people who claim social security benefits^47^ ^48^ or research into the impact of mandatory positive affect on an expanding range of ‘unproductive’ or failing citizens:^16^ those who are out of work, not working enough, not earning enough and/or failing to seek work with sufficient application.

A number of reports produced for the Cabinet Office under both the previous Labour government and the current Coalition have drawn centrally upon psychology and behavioural economics for the legitimation and direction of behaviour change policy or ‘instrumental behaviourism’.^49–51^ The mission of the Cabinet's Behavioural Insights Team or ‘nudge unit’—‘the application of behavioural science and psychology to public policy’—is a recent statement in this tradition.^52^ The psychological sciences in combination with behavioural economics provide both an ostensibly scientific model and the means for a positive self-image for policymakers and practitioners within the welfare-to-work sector. This notion has considerable traction so that even critics of recent UK government active labour market policies who advocate the abolition of benefit sanctions suggest that

insofar as it is desirable to attempt to influence claimants’ behaviour […] this should be done through a scientific approach.^53^

Psychology allied to behavioural economics allows the sector to consolidate its self-conception as an industry in its own right that sets its own standards and regulates itself via the Employment Related Services Association (established 2005) and the Institute of Employability Professionals (launched 2012).^54^ ^55^

In this setting, psychology (and ‘therapy discourse’ more generally) coproduces and validates the core mythologies of neoliberalism, while simultaneously undermining and eroding alternative discourses—of solidarity, collectivity and interdependence.^56^ It functions not only to reinforce the view that achieving the status of (paid) working citizen is ‘the pinnacle of human experience’ (p.29)^23^ but also to construct a very specific definition of the attitudes, beliefs and attributes that constitute ‘employability’: the ‘right kind of subject’;^57^ the ‘right kind of affect’.^47^ The roll-call of valued characteristics familiar from positive psychology, the wellbeing industry and public health—‘confidence, optimism, self-efficacy, aspiration’—are imposed in and through programmes of mandatory training and job preparation. They also feature centrally in the way in which people receiving benefits frame their own experiences.^58^ The duties of citizenship are expanded to include enforced rational self-governance so that liberal subjects’ capabilities, inclinations and desires are in accord with values and expectations that are identified as already given by a civil society centred on the labour market.^13^ ^14^ For example, in Labour MP Graham Allen's 2011 report on early intervention public health and education policy, ‘life readiness’ is said to consist in

having the social and emotional capability to enter the labour market; understanding the importance and the social, health and emotional benefits of entering work, the impacts of drug and alcohol misuse, crime and domestic and other violence (p.9).^59^

These kinds of policies, seeking to model in unemployed people the imperatives of the market, are carried out by means of the market, through those who are paid to ‘activate’ claimants and those who benefit from their unpaid labour.

## Positive affect

The growth and influence of discourses of positive affect in these and other systems of governance and ‘technologies of the self’ has been widely observed.^16–18^ ^21^ ^32^ ^47^ ^48^ ‘Strengths-based discourse’ is a significant policy imperative in both health and welfare reform. Positive affect plays an important supporting role in policy preoccupations with how best to manage the intersection of long-term conditions and long-term unemployment, exemplified in the shift from rest cure (signified by the sick note), to work cure (signified by the fit note).^60^

The psychological attributes and dispositions of individuals and communities (the ostensible presence or absence of optimism, aspiration, self-efficacy, conscientiousness, sense of coherence) are being used to account for unemployment (and for a range of other social outcomes, notably health inequalities) and are promoted via psychological interventions that aim to modify cognitive function or emotional disposition/affect.^61^ Signing up for these interventions is an explicit or implicit condition for receiving support. These trends intersect with and are reinforced by the parallel rise in brain science—‘reading social problems through understanding the brain’—which correlates outcomes (crime, addiction, health behaviour, educational attainment) with brain structure.^62^ ^63^ Conditions of psychological deficit are both scientifically and medically legitimised. A cheerful disposition, in combination with a thankful heart and highly developed ‘executive control’, is so widely celebrated in the policy literature that the politics of this reification are rarely questioned.^64^ These developments may help to explain what lies behind the marked decline in solidarity with unemployed citizens and welfare claimants and the heightened stigma in daily life and public discourse experienced by people who are poor.^65^ They also tend to preclude acknowledgement of the corporate and charitable sector beneficiaries of workfare and the ‘low pay, no pay’ economy that workfare supports, or the estimated £25 billion per annum paid in benefits to workers receiving wages below subsistence levels.^44^ ^66^

## Boycott workfare: history of a campaign

While there is considerable evidence of this hardening of public attitudes towards benefit claimants, the value of mandatory unpaid work activity and enforced ‘volunteering’ is strongly contested. There are numerous campaigning and claimant solidarity groups in the UK and the rest of Europe whose activities are concentrated in this area. One is Boycott Workfare, which evolved through the work of people who have experienced workfare in the UK. Formed in 2010, it is a movement that campaigns against the imposition of forced, unpaid work on several levels: by taking action to expose the involvement of companies and other entities in taking or arranging placements or providing mandatory training, and by acting as a point of information for claimants and other claimants’ organisations:

We expose and take action against companies and organisations profiting from workfare; encourage organisations to pledge to boycott it; and actively inform people of their rights.^67^

Informing people of their rights means proposing a model of activity opposed to and subversive of the ‘activated’ welfare subject.

Undoing the legitimacy conferred on workfare, in part by its association with psychology, is a central concern of the campaign, as is counteracting the variously inflected negative stereotype of unemployed people. The ‘naming and shaming’ of organisations participating in workfare has led large numbers to withdraw and is a central factor in DWP efforts not to publish names of those involved. For example, the DWP argued (in appealing the Information Commissioner's decision that they must publish the names of companies involved in Mandatory Work Activity) that making this information public “would have been likely to have led to the collapse of the […] scheme”.^68^ ^69^ Concerns that mandatory placements undermine the meaning of volunteering have also led many voluntary agencies to sign a ‘keep volunteering voluntary’ agreement, undertaking not to take part in workfare schemes.^70^

## Waiting for a wage

It is important to understand the extent to which activities that until very recently would have been classified as ‘work’ are now rebranded as ‘work preparation’ and are hence both unpaid and characterised in terms of ‘psychological preparation’. The Apprenticeship and Traineeship Database reveals the very wide range of private sector organisations offering ‘unpaid’ opportunities—which are seen to enable young people to become ‘work ready’, an attribute that is essentially about ‘motivation’ and the ‘right attitude’ to work. Tasks that would once have provided paid Saturday or holiday jobs for young people are now provided free of charge to major employers, often in the absence of any return, apart from an ‘exit interview’. One unpaid traineeship opportunity lists the following tasks:

check and top up under bonnet levels on a vehicle; check anti-freeze content and recommend action; check and adjust tyre pressures; fit a standard light vehicle tyre; balance steel and alloy wheels; change oil and filter; replace spark plugs on a 4 cylinder engine; replace an air filter; torque up wheel nuts to the correct settings.^71^

There are currently around 50 traineeships on offer in the National Health Service where for no pay you can do

administration and reception work; hospitality and catering; service areas, including portering and post; assisting in clinical areas.^71^

One gets little in return for working unpaid 4 days per week, for 30 h, for up to 6 months in any of these rebranded jobs:

We expect all traineeships to offer a guaranteed interview with the work placement host at the end of the placement. Where possible, the young person should receive a real job interview where a post or apprenticeship has become available. However, we recognise that this will not always be feasible and in these cases a formal exit interview with the employer who provided their work placement will help the young person to practice and prepare for future opportunities (p.13).^72^

Like workfare, traineeships contribute to the separation of work activity from wages. An unemployed person creates value and generates income for everyone except themselves. Recent developments show that ‘waiting for a wage’ has been extended to job applicants, with some employers requiring applicants to undertake ‘voluntary shifts’ before receiving a job offer: “I had interview in May for Events job. They wanted me to work 2 week trial for free! UNPAID! 8.30am/10pm” (comment by Shaun, on Thomson).^73^

## The lived experience of workfare

The imposition of psychological explanations for unemployment functions to erase the economic realities of the labour market and authorises the extension of state-sanctioned surveillance to psychological characteristics. Compulsory positive affect and psychological authority are being applied in workfare in order to (1) identify ostensible psychological barriers to gaining employment and to inculcate attributes and attitudes said to increase employability; (2) punish people for non-compliance (through conditionality and benefit sanctions) and (3) legitimise workfare and other coercive labour market measures.

These developments mean that positive psychology is now as significant a feature of conditionality in the lives of those who are poor as going to church once was, and they share a common evangelical language: “something within the spirit of individuals living within deprived communities that needs healed”.^74^ Unfortunately, the compulsions of positive affect are not confined to Sundays.

I am shy and have difficulty speaking to people and I will not do play acting in front of a group of people I am very uncomfortable with […] I was told I would be sanctioned if I didn't take part, so I said I would get up, but I am not speaking […] After that, we had to fill out yet another ‘benefits of being assertive’ sheet.^75^

The consistent failure of workfare interventions to achieve their stated aim of improving work outcomes—both in the UK and internationally—has resulted in a much greater focus on psychological or ‘soft outcomes’, said to ‘move people closer to work’.^38–42^ ^76^ ^77^ A 2012 evaluation of an ineffective three-stranded scheme, on which DWP's recent ‘Help to Work’ three-part programme is based, found that^78^ ^79^

while there was no significant difference in job outcomes at the end of the programme the OCM^iv^ and CAP^v^ trailblazer strands were successful in achieving soft outcomes such as increases in motivation, confidence, job-seeking behaviour and a positive change in attitudes to work. These softer impacts may yet translate into job outcomes and sign off from JSA (p.4).^80^

‘Soft outcomes’ disarticulate work and wages by treating a job as something that may be gained by possessing the right attitude to work (an attitude for which one must labour) and work as something to be valued because it evinces and activates the right attitude in the (potential) employee—rather than because it allows one to purchase a living. At the same time, the means by which soft outcomes are regulated (sanctions: for failures in attitude and in compliance with the actions demanded by active labour market measures) link together more closely than ever a person's failure to manifest the right attitude and their inability to afford to purchase a living.

Efforts to achieve these ‘soft outcomes’ are evident in the course content of mandatory training programmes run by major workfare contractors like A4e and Ingeus and are increasingly apparent in the personal testimonies of claimants:

I've been claiming Jobseeker’s Allowance for about 8 weeks. I haven’t sworn or shouted at anyone. I have had 3 advisor interviews already; yesterday my adviser asked me to see their psychologist. I did not consent. I've been told that I shouldn't look into things too deeply...& that I am asking too many questions.^81^

The choice was to accept psych eval, or go straight to MWA.^81^

You've got all these hooks on you…it's your way of being…you need to shift the way you look at it. You've got all this anger and frustration and that's stopping you from getting a job. It comes across in your CV.^82^

I duly attended the offices of A4e and (along with 6 other “customers”) was treated to INSPIRE. This turned out to be a session on Neuro Linguistic Programming (NLP) run by an outside company claiming to be “Master Practitioners in NLP”. I was “mandated” to attend under threat of loss of benefits and was effectively unable to leave the session because of the same ever present threat.^83^

My ‘advisor’ said I needed to see a psychologist because I was tearful and anxious after having my JSA cut for 4 weeks despite having a young child to look after by myself. When I said I did not trust anyone who finds it acceptable to starve others as a punishment, he told me that I was paranoid and again, needed to see a psychologist.^84^

The A4e Engage Module states: “students will learn how to develop the right mindset which will appeal to employers” (other elements of this module are assertiveness, confidence, benefits of work, motivation and enhance your mood). As Esther McVey, Minister of State for Employment, announced recently, jobseekers are expected to take steps to make themselves attractive to employers: “employers looking to fill vacancies want people who are prepared, enthusiastic and job-ready”.^85^ Willingness to submit to coerced labour becomes an index of the (approved) disposition and beliefs possessed by an unemployed person.

Izzy Koksal, in her blog on the experience of A4e training, describes the impact of being surrounded by motivational quotes, with their persistent emphasis on individual responsibility for unemployment and the perils of negative thinking.^82^ A sheet full of affirmations, handed out to participants in the ‘confidence building’ workshops that form part of Ingeus’ delivery of the Work Programme, include such motivational statements as

Go hard, or go home

My only limitations are the ones I set for myself

Failure is the path of least persistence

Success is getting up one more time than you fall down

It's always too soon to quit

Nobody ever drowned in sweat

The sin isn't falling down but staying down

No one can make you feel inferior without your consent^86^

People have described feelings of anger, humiliation and depression on receiving daily ‘positive’ emails from welfare to work contractors such as A4e: “success is the only option”; “we're getting there”; “smile at life”; “this can be the greatest, most fulfilling day you've ever known. For that to happen, you have to allow it” (Warren Clark, personal communication, 2013).^87^

Reflecting on the feedback they received from Learn Direct (a major training provider), following a 4-week unpaid placement at the Salvation Army, one person wrote:

attitude to work….no idea why they rated me poor for this, I was willing to work, I travelled by train every day then walked a long walk from Edinburgh station to the store every single day for 4 weeks!, and done everything asked plus more!^88^

He is concerned because deficits in attitude and motivation can and do trigger sanctions. Psycho-coercion of this kind is directly contributing to the escalation of the number of sanctions being applied, forcing people off benefits and plunging growing numbers into poverty:^28^ ^42–44^ ^53^ ^89^ ^90^ eligibility for both out-of-work and in-work benefits is contingent not only on certain behaviours but also on possession of positive affect; conditionality is linked to the ‘employability’ mindset. For example, one of the criteria for being sent on Community Work Placements (unpaid work for 30 h per week, for 26 weeks) is ‘lack of motivation’, although this is never defined.^91^

The messages in the course handout for Ingeus’ mandatory ‘Healthy Attitudes for Living’ course take these themes a step further, intended, perhaps, to counter any residual yearnings in the jobseeker for either justice or security and to pre-empt reflection on the social gradient in ‘bad things happening’:

Sometimes life's just plain unfair. Bad things happen to the nicest of people. On top of being unfair, life's unpredictable and uncertain a great deal of the time. And really, that's just the way life is […] If you can accept the cold hard reality of injustice and uncertainty, you're far more likely to bounce back when life slaps you in the face. You're also less likely to be anxious about making decisions and taking risks. But remember, you can still strive to play fair yourself.^86^

This Ingeus module argues that one “common thinking trap” is “catastrophising”: “you may exaggerate or magnify the negative aspect of an event”; “you may view the probability of disaster as great”. One is encouraged to “[recognise] the negative thinking error” and take “calculated risks”.^92^ Of course, power over certain catastrophes lies with Ingeus staff, who are responsible for raising a ‘compliance doubt’ against an unemployed person, the first step towards being sanctioned. In addition to mandatory training informed by positive psychology, claimants may also be subjected to strengths-based interventions, including online psychometric testing—‘failure to comply may result in loss of benefits’.^93^ And as Cromby and Willis have noted,^48^ every aspect of the Values in Action ‘Inventory of Signature Strengths’ test recently imposed on claimants contravened the British Psychological Society's (BPS) ethical code.

Working on psychological deficits becomes the full-time, unpaid labour of millions of people,^vi^ which, together with mandatory job search activities, ensures that these days people who are poor have no money, no time—and no place:

Basically what I'm saying in short is that I feel there is no place in society for a quiet, shy, creative person like me. And now I feel I don't even deserve to call myself creative, because I don't even do that anymore, because I am too depressed.^94^

In a scheme recently announced, claimants will undergo interviews to assess whether they have a ‘psychological resistance’ to work, along with attitude profiling to judge whether they are ‘bewildered, despondent or determined’.^79^ Those deemed ‘less mentally fit’ will be subject to more intensive coaching, while those who are ‘optimistic’—such as graduates or those who have recently been made redundant—can be placed on less rigorous regimes. This classification system will be used to recruit to a new scheme obliging those who are long-term unemployed to spend 35 h a week at a job centre.

The context in which positive psychology's motivational techniques are deployed, then, is one structured by a regime of tacit and explicit threat and coercion, in which one can never be sure whether or not a sanction will be tagged to a particular instance of behaviour or attitude. As many first-hand accounts witness, Jobcentres and the premises of welfare-to-work contractors are not neutral settings for interventions or decisions about the relative degree of unemployed people's material hardship, ‘willingness to work’, ‘readiness’ for work or ‘resistance’ to work: they are intensely anxiety-inducing and intimidating locations that bear witness to marked imbalances of power.^95–97^

What is perhaps more noteworthy than all these developments is the response of the professional body responsible for ethics and accountability of psychology and psychologists. BPS has confined itself to saying that such tests must be administered by experienced users of psychometrics under supervision of a chartered psychologist.^98^

## Conclusions

[T]he voices of resistance against the abjectifying logics of neo-liberal governmentality are growing louder (p.2).^65^

The participation of psychology and psychologists in the delivery of coercive goals in welfare reform clearly raises ethical questions. As Wright (p.2)^20^ has observed, “the active welfare subject is a figure of aspiration, a transformation possible only via coerced self improvement”. Psychology now plays a central and formative role in stigmatising the ‘existence and behaviour of various categories of poor citizens’ (p.9)^99^ and in legitimating the measures taken to transform and activate them. Personality, disposition and behaviour are abstracted from context, history and political struggle, obscuring the fact that the distinction between those with appropriate levels of ‘optimism’ and those without is essentially a class distinction.^80^ Mandatory work-related activity and ‘supported job searches’ involve tasks experienced as humiliating and pointless by jobseekers:^75^ ^94^ the ‘grotesque daily practices of condemnation and disenfranchisement’ that contribute to the social abjection of the most socially and economically disadvantaged citizens (pp.170–1).^65^ There is no evidence that work programme psycho-interventions increase the likelihood of gaining paid work that lasts any length of time. In perpetuating notions of psychological failure, they shift attention away from the social patterning of unemployment and from wider trends: market failure, precarity, the rise of in-work poverty, the cost of living crisis and the scale of income inequalities.^44^ ^46^ They contribute centrally to the reification of paid work and the concomitant devaluing and discounting of all other activities, contributions, values and commitments. Above all, psychology is implicated in what amounts to a ‘substitution of outcomes’, where the modification of psychological attributes stands in for delivering actual improvements in household income or increasing the availability of real paid work.

## Resistance

In the reification of positive affect, what is absent is any reference to the contested nature of constructs such as personality and attitude, their ideological underpinnings and the processes through which specific characteristics or attributes acquire both social value and economic reward. In other words, the political nature of these issues is evaded.^61^ Psychological fundamentalism—also evident in the burgeoning well-being industry—together with the rise of psychological conditionality, has a very direct impact on the lives of people claiming welfare benefits. This impact has barely been documented and highlights the need for deeper research scrutiny and more pressing questions about relationships between psychology and the medical humanities. The ‘black boxing’ to which we have referred also means that—for both political and methodological reasons—independent research is especially important in tracing and making transparent the confluence between medico-corporate interests and manifold forms of labour market governance.

Even so, these questions are being asked elsewhere, in the emergence of multiple forms of resistance to neoliberal definitions of value and worth and to the erosion of hard-won rights of social citizenship. Workfare has become an important site for satire on the fetishisation of paid work, for struggle over definitions of a meaningful and productive life and for attempts to embrace myriad shades of human experience and human subjectivities, with notable contributions from those whose welfare dependency is most decried.^64^ ^100–102^ The disability rights movement has played a central role in challenging the discourse of ‘no legitimate dependency’ and in using direct action to express solidarity and to forge discourses and practices that can shape positive identities for people claiming social security.^56^ As the coercive use of positive affect in workfare demonstrates, there are good reasons to prefer the politics of rights and justice to the discourses of positive psychology.

## Footnotes

[i] For example, eligibility to receive cheap food from community shops (‘inspiring motivation and confidence in our members’) requires beneficiaries to be motivated to make positive change in their lives and to sign up to a personal and professional development programme, the ‘success plan’^4^

[ii] Medical humanities is an emerging field. Some of the central themes of enquiry relevant to our concern with the use of psychology to discipline citizens are explored through the work of the Centre for Medical Humanities in Durham (https://www.dur.ac.uk/cmh/themesofenquiry/mindbodyaffect/).

[iii] Social media are a primary source of personal accounts of the experience of psychological coercion; Twitter Facebook, the comments section of online articles and blog platforms represent some of the few opportunities for claimants to speak out about the content of mandatory training courses, psychological ‘referrals’, receiving daily ‘positive’ emails and the prevalence of positive psychology ‘messaging’ by Jobcentres and welfare-to-work subcontractors. Boycott Workfare also receives personal testimonies via email and the ‘name and shame’ forms submitted to the website (identifying business and charity users of workfare schemes). We deal with these forms of personal document in the course of our activities with Boycott Workfare; they can be made public where permission has been granted from the person involved. We have also sought permission before citing experiences detailed in blogs and other social media accounts. The ephemeral nature of social media, the use of pseudonyms, claimants’ fears of sanctions and retribution if identified and the painful nature of many of the experiences described raise a number of difficulties in accessing, collecting and selecting these data. Addressing these challenges is an important future research agenda for the medical humanities and forms part of a project on workfare at the Wellcome Trust.^21^

[iv] Ongoing Case Management: more intense Jobcentre-based surveillance, coupled with a range of mandatory activities.

[v] Community Action Plan: a 6-month-long workfare placement, coupled with supported job search (identical to the Community Work Placement strand of the current ‘Help to Work’ scheme).

[vi] More than 1.6 million people had joined the Work Programme alone as of June 2014.^40^
